# Effect of physical exercise on sleep quality in college students: Mediating role of smartphone use

**DOI:** 10.1371/journal.pone.0288226

**Published:** 2023-11-03

**Authors:** Chuan-Yi Xu, Kai-Tuo Zhu, Xiang-yan Ruan, Xiao-Ya Zhu, Yang-Sheng Zhang, Wen-Xia Tong, Bo Li

**Affiliations:** 1 Ministry of Sports, Guangxi University of Chinese Medicine, Nanning China; 2 Graduate School, Kyonggi University, Suwon City, Korea; 3 Physical Education College, Shangqiu University, Shangqiu, China; 4 School of Physical Education, Nanjing Xiao zhuang University, Nanjing, China; 5 Physical Education College, Yangzhou University, Yangzhou, China; 6 Institute of Sports Science, Nantong University, Nantong, China; University of Study of Bari Aldo Moro, ITALY

## Abstract

**Objective:**

To investigate the effect of physical exercise on sleep quality and the mediating effect of smartphone use behavior in college students.

**Methods:**

A cross-sectional study design was adopted. An online survey of 5,075 college students was conducted using the Physical Activity Rating Scale-3, the Pittsburgh Sleep Quality Index, and the Mobile Phone Addiction Tendency Scale.

**Results:**

The sleep quality of college students was poor, and the proportion of college students with good sleep quality was 23.567%. A significant correlation existed between sleep quality and physical exercise (r = −0.159, ***P*** < 0.001) and mobile phone addiction (r = 0.355, ***P*** < 0.001). Physical exercise can predict sleep quality in college students (β = −0.011, ***P*** < 0.001). Smartphone use plays a part in mediating the process by which physical exercise affects sleep quality.

**Conclusion:**

Chinese college students have poor sleep quality. Physical exercise and smartphone use behavior are important factors affecting the sleep quality of college students. Physical exercise can directly predict the sleep quality of college students and can predict the sleep quality of college students through the mediating effect of smartphone use behavior.

## 1 Background

There are 40 million university students in China, and the physical and mental health of the university student population has always been an important concern for the Chinese government and scholars. Sleep is an indispensable physiological phenomenon for human beings. Sleep is a healing process in the human body that restores the spirit and relieves fatigue [1]. Approximately 33.33% of the entire life of human beings is spent in sleep; meanwhile, good sleep is one of the three health standards recognized by the international community, and extremely short sleep or poor sleep will affect health [1–4]. The harm of insomnia and long-term irregular sleep is enormous. A new study showed that in a student population, students who slept less than 9 hours a night had smaller brain regions responsible for memory, intelligence and well-being than those who slept healthy each night, and two years later, these differences persisted, meaning that sleep-deprived people suffer long-term damage to their brains [2]. Sleep quality is clearly one of the important constraints of human health.

In China, more than 300 million people have sleep disorders, the incidence of insomnia in adults is as high as 38.2%, and more than 90% of primary school students do not sleep up to the standard, and these data are increasing yearly [5]. In particular, due to the COVID-19 pandemic, Chinese universities have implemented “normalized” closed management [6]. College students’ daily recreational, socializing, and physical activity activities have decreased, and the use of electronic products, such as mobile phones, and addiction rates have increased [7, 8]. This phenomenon may lead to a decline in sleep quality [9, 10], which will directly affect the physical health and learning efficiency of college students. Therefore, after the current Chinese government downgraded COVID-19 to “category B,” how to efficiently improve the sleep quality of college students is an important issue facing scholars at present.

The length of sleep in humans is related to social timing, light and dark exposure, genetic factors, gender, and developmental age [4]. Among them, sports are favored by scholars for their cost-effectiveness as a research subject and their ability to function as a good sleep aid [11, 12]. The possible mechanism by which exercise helps sleep is that exercise intensity causes physical exhaustion, and the brain’s exhaustion response to the body is to increase deep sleep time. Moreover, aerobic exercise can avoid the flattening of the body temperature rhythm curve, and the peak temperature of the person after exercise is at a high level; however, as the temperature lowers, it can reach a level much lower than before, and the body temperature drops. As people will eventually feel sleepy after exercise, they will experience better sleep at night [13]. Therefore, in recent years, relevant studies have also attempted to observe the sleep quality of participants through different exercise interventions, which is a good exploration.

Mobile phone addiction caused by smartphone use not only reduces sleep quality and leads to negative emotions such as burnout and procrastination [14–16] but also increases screen time and sedentary behavior and directly and indirectly interferes with college students’ various forms of physical activity [14, 17–19]. Obesity, cardiovascular disease, and decreased immunity attributed to decreased physical activity are also evident [20, 21]. Smartphone use has shown strong penetration and impact among college students [22]. College student mobile phone addicts are easy to indulge in various activities (network information and technical operations, among others) mediated by mobile phones, their physical activity time, opportunities and resources will be occupied and replaced by a large amount of screen time or sedentary behaviors [23, 24]. Therefore, mobile phone addicts are often accompanied by weak exercise motivation and interest apart from “low frequency, short holding time, small intensity” and other exercise characteristics; furthermore, their physical activity can hardly reach the ideal recommended amount standard [20]. Mobile phone addiction leads to increased sedentary behavior and decreased physical activity, and vice versa. This vicious cycle can severely reduce the quality of sleep of college students. The prevalence of poor sleep quality due to smartphone use has increased significantly, particularly during the COVID-19 pandemic [25, 26].

On the basis of the aforementioned literature review, this study examines the effects of physical exercise and smartphone use behavior on sleep quality in college students through the research paradigm of a cross-sectional survey. The current situation of the sleep quality of college students was discussed from the level of “influencing factors.” The advantage of this study is that the current status of physical exercise behavior and sleep quality of Chinese college students can be explained through a large-scale survey. The corresponding research results can provide data support for the development of policies to promote the physical and mental health of university students after the COVID-19 pandemic has ended.

## 2 Research methods

A cross-sectional study design was used in this study. In the collection of data, the online questionnaire was used uniformly. All the scales are already widely used, and all have been tested for reliability and validity in Chinese college students. In this study, SPSS 25.0 software was used for data processing.

### 2.1 Object

Stratified random sampling was used. In September 2022, a questionnaire survey was conducted among college students in three universities, Shangqiu University in Shangqiu City, Henan Province, Guangxi University of Traditional Chinese Medicine in Nanning City, Guangxi Zhuang Autonomous Region, and Nanjing Xiaozhuang College in Nanjing, Jiangsu Province, and 5,199 questionnaires were distributed according to the administrative class, of which 5,075 questionnaires were effective with an effective rate of 97.61%. The sample size in this research meets the minimum sample size requirement. The distribution of study subjects is shown in Table 1.

**Table 1:** Distribution of research objects.

		Frequency	Percentage
Gender			
	Male	2031	40.020
	Female	3044	59.980
Grade			
	1	1815	35.764
	2	2292	45.163
	3	589	11.606
	4	379	7.468
Total			
		5075	100

The minimum sample size calculation is performed using Eq (A) [27]. Class I error α is set to 0.05, the allowable error δ is set to 0.01, and the sample rate ρ is set to 0.05. After checking the official websites of each university, the total number of undergraduate students in the three universities is 98,930 (updated in 2022), and the limited total number N is set at 98,930. After calculation, the minimum sample size for this study was n = 1792 people.


$$n=\frac{\frac{z_{\alpha }{}^{2}*\sigma^{2}}{\delta^{2}}}{1+\frac{z_{\alpha }{}^{2}*\sigma^{2}}{\delta^{2}}/N}$$ (A)

### 2.2 Scales

In addition to basic sociodemographic information, the online questionnaire used by this institute mainly comprises the following three scales: Physical Activity Rating Scale-3 (PARS-3) for measuring the physical activity behavior of college students, the Pittsburgh Sleep Quality Index (PSQI) for measuring sleep quality, and the Mobile Phone Addiction Tendency Scale (MPATS) for measuring the smartphone use behavior of college students.

### 2.2.1 PARS-3

PARS-3 was compiled by the Japanese scholar Kōo Hashimoto and revised by the Chinese scholar Liang [28]. PARS-3 examined the amount of physical activity from three aspects: the intensity and frequency of physical exercise and the time of a single exercise, and used this to measure the participants’ participation in physical exercise. In the specific questionnaire completion, each question item is divided into 5 levels, and the score is 1‒5. The raw score from the questionnaire measurement is calculated using formula (B).

### *Physical exercise volume score = intensity × (time– 1) × frequency*. (B)

The norm rating standard for Chinese adults of PARS-3 is 1≤9 points for small exercise, 20‒42 points for medium exercise, and ≥43 points for large exercise [28]. The results of the PARS-3 represent a measure of the amount of physical activity of the participants, which to some extent reflects the current status of college students’ sports participation behavior at a specific time.

#### 2.2.2 PSQI

The PSQI was developed in 1989 by Dr. Buysse, a psychiatrist at the University of Pittsburgh and others, to measure the sleep of adults in a month [29]. Chinese scholars Liu and Lu successively tested the reliability and validity of the PSQI in college students and confirmed that the PSQI has good reliability and validity in college students [30, 31]. The PSQI is used to evaluate the quality of sleep of the participants in the past 1 month, including sleep quality, sleep onset time, sleep time, sleep efficiency, sleep disorders, hypnotic drugs, and daytime dysfunction in seven aspects. Each component is scored according to grades 0‒3. The cumulative score of each component is the total score of the PSQI, and the total score range is 0‒21. The higher the score is, the worse the sleep quality. The Chinese adult sleep quality score of the PSQI is normal: 0‒5 sleep quality is very good, 6‒10 sleep quality is not bad, 11‒15 sleep quality is poor, and 16‒21 sleep quality is very poor. In this study, the retest reliability of the PSQI was 0.994, the fractional confidence coefficient was 0.824, and the overall Cronbach’s α coefficient was 0.845.

#### 2.2.3 MPATS

MPATS was compiled by Chinese scholar Xiong [32]. MPATS adopts the Likert five-point scoring method, which is 1‒5 points from complete nonconformity, not very conformant, average, somewhat compliant, and completely compliant. The highest score of the MPATS is 80 points, and the lowest score is 16 points. The higher the score is, the greater the tendency to use mobile phones, and vice versa (i.e., the lower the addictive tendencies). In terms of reliability and validity used by Chinese college students, the Cronbach’s α coefficient of the MPATS is 0.83, and the α coefficient of 4 factors is between 0.55‒0.80. MPATS has a retest reliability of 0.91. At present, the evaluation model of MPATS’s college student group is still being conceptualized.

### 2.3 Quality control

The quality control of the research included the following aspects: The unification of the research plan and the implementation of the questionnaire survey, special training for the investigators in the early stage of the formal survey, the development of standardized introduction language, the proficiency of the content of the questionnaire, and the correctness of the questionnaire filling. The investigators were college counselors. In the data preprocessing, data such as logical errors and omissions were retested or eliminated to ensure the authenticity and validity of the data. Statistical data processing requirements were strictly followed, corresponding statistical methods were used for different types of data.

### 2.4 Statistical analysis

Statistical analysis of data was performed using SPSS 25.0 software. The statistical steps for the main results were as follows: first, the descriptive data analyzed the current status of physical activity behavior and sleep quality of college students, and the chi-square test was used to analyze the differences in physical activity behavior and sleep quality of college students of different genders and grades (Cramer’s V coefficient was used for the effect amount). Second, correlation analysis was used to examine the correlation between three indicators: smartphone use, sleep quality, and physical activity behavior. Finally, linear regression analysis was used to verify the mediating effect of smartphone use on the prediction of sleep quality by physical exercise behavior, and the three variables were normalized (Z score) before the mediation effect test.

## 3 Results

### 3.1 Descriptive analytics

As shown in Table 2, the proportion of college students with “very good” sleep quality was low, accounting for only 23.567% of the total. The proportion of “very poor” sleep quality accounts for 9.34% of the total, and this part of college students may be a group of sleep disorders (e.g., insomnia and nightmares, among others), which needs attention. By contrast, large differences in sleep quality were apparent between gender (P < 0.001, Cramer’s V = 0.116) and grade (P < 0.001, Cramer’s V = 0.138). From the number of people in this group with “very good” sleep quality, the sleep quality of male college students is better than that of female college students. First-graders had the best sleep quality, and fourth-graders had the worst sleep quality.

**Table 2:** Basic results of tests of physical exercise and sleep quality in university students.

Index		Overall		Gender				Grade							
				Male (n = 2031)		Female (n = 3044)		1 (n = 1815)		2 (n = 2292)		3 (n = 589)		4 (n = 379)	
		n	%	n	%	n	%	n	%	n	%	n	%	n	%
Sleep quality															
	Very good	1196	23.567	577	28.410	619	20.335	498	27.438	490	21.379	135	22.920	73	19.261
	Not bad	2262	44.571	803	39.537	1459	47.930	865	47.658	1004	43.805	222	37.691	171	45.119
	Poor	1143	22.522	425	20.926	718	23.587	357	19.669	528	23.037	162	27.504	96	25.330
	Very poor	474	9.340	226	11.128	248	8.147	95	5.234	270	11.780	70	11.885	39	10.290
	χ^2^			68.370				96.214							
	p			<0.001				<0.001							
	Cramer’s V			0.116				0.138							
Physical exercise															
	Minimal	3863	76.118	1208	59.478	2655	87.221	1367	75.317	1776	77.487	434	73.684	286	75.462
	Moderate	693	13.655	391	19.252	302	9.921	277	15.262	279	12.173	80	13.582	57	15.040
	Active	519	10.227	432	21.270	87	2.858	171	9.421	237	10.340	75	12.733	36	9.499
	χ^2^			604.672				18.846							
	p			<0.001				<0.001							
	Cramer’s V			0.345				0.052							

Physical exercise behavior showed that 76.118% of college students exercised “small,” and only 10.277% of college students exercised “large.” The physical exercise behavior between male and female college students (P < 0.001, Cramer’s V = 0.345) also significantly differed, and the proportion of moderate exercise and heavy exercise among male college students was higher than that of female college students. In terms of grades, the overall exercise of third-graders is better than that of other grades. Physical activity among college students presented a grade difference (P < 0.001, Cramer’s V = 0.052).

### 3.2 Correlation analysis

The results of correlation analysis showed that the sleep quality level of college students was significantly negatively correlated with the level of physical activity (r = −0.159, P < 0.001) (Table 3). In particular, the higher the PARS-3 score (i.e., the more active the physical activity), the lower the PSQI score (i.e., the better the sleep quality). The sleep quality level of college students was positively correlated with the total mobile phone addiction score. In particular, the higher the MPATS score (i.e., the more active the behavior of smartphone use or the greater the tendency of mobile phone addiction), the higher the PSQI score (i.e., the worse the sleep quality). At the same time, the results of the correlation analysis showed that the physical activity level of college students was significantly negatively correlated with the total mobile phone addiction score, and the abovementioned results showed that the three variables can be further analyzed for mediating effects.

**Table 3:** Correlation analysis.

	Sleep quality	Physical exercise	Mobile phone use
Sleep quality		−0.159^※※^	0.355^※※^
Physical exercise	−0.159^※※^		−0.165^※※^
Mobile phone use	0.355^※※^	−0.165^※※^	

**. At level 0.01 (double-tailed), the correlation is significant.

### 3.3 Mediation effect test

The test of variance in regression analysis showed that the P values were all less than 0.05, and the regression model was valid (Table 4). The regression results of Eq (1) showed that physical exercise behavior significantly predicted the sleep quality of college students (***β*** = −0.011, P = 0.001). In particular, the higher the PARS-3 score was, the lower the PSQI score. In other words, the better the sleep quality of students who participate in more physical activity. The regression results of Eq (2) showed that mobile phone addiction can significantly predict the sleep of college students (***β*** = 0.397, P < 0.001). In particular, the higher the MPATS score, the higher the PSOI score. In other words, the more serious the mobile phone addiction of college students, the worse their sleep quality. Overall, the prediction of sleep and mobile phone addiction tendency by physical exercise reached a significant level. Eq (1) found that physical exercise explained 1.1% of sleep variation, and when the mobile phone addiction variable in Eq (3) intervened, the variation of physical exercise to sleep increased to 1.6%. At the same time, the regression coefficient of mobile phone addiction to sleep decreased from 0.397 in Eq (2) to 0.396 in Eq (3). The data suggest that mobile phone addiction has a partial mediating effect between physical exercise and sleep quality.

**Table 4:** Analysis of the mediation effects.

Equation	Independent variable	Dependent variable	Model Summary		ANOVA		Coefficient		
			R^2^	Adjusted R^2^	F	*p*	β	t	*p*
(1)	Physical exercise	Sleep	0.002	0.002	11.958	0.001	−0.011	−3.458	0.001
(2)	Mobile phone use	Sleep	0.158	0.158	950.875	<0.001	0.397	30.836	<0.001
(3)	physical exercise and Mobile phone use	Sleep	0.158	0.158	476.219	<0.001	−0.016	−1.214	0.025
							0.396	30.631	<0.001

## 4 Discussion

Sleep quality problems are common among the health problems of Chinese college students and are a huge challenge to Chinese university administrators and scholars who study the psychology of college students. This study verifies the effect of physical exercise on sleep quality in college students from an empirical perspective and introduces the mediating variable smartphone use behavior. This study attempts to analyze the current status of physical exercise, sleep quality and smartphone use among Chinese college students from the perspective of “influencing factors.” The results of the study will provide a data reference for focusing on improving the sleep quality of college students in the “post-epidemic era.”

### 4.1 Problem of sleep quality in college students needs attention

The results of this study show that the sleep quality of college students is “very good” in only 23.567% of the total. The American Academy of Sleep Medicine’s “Healthy Sleep Habits” states that adults over the age of 18 should sleep sufficiently for eight hours a day and need to maintain good “sleep hygiene” [33]. Previous studies have shown that sleep quality in Chinese college students is worrying, with a prevalence of 39.4% in 5001 college students in a cross-sectional study of Hong Kong, China [34]. Chinese mainland sleep quality data were similarly poor among college students [35, 36]. In addition, the results of this study are consistent with the data in the China Sleep Research Report (2022) released by the Chinese government. According to the data of the “China Sleep Research Report (2022),” nearly 75% of the participants had sleep problems, and difficulty falling asleep became the number one problem. Compared with different age groups, young people stay up late more, and elderly individuals cannot sleep. More than 75% of young people aged 19‒25 stay up until after midnight, which is a well-deserved “staying up champion.” Young adults aged 19‒35 is the age group with a high incidence of sleep problems, and poor sleep has gradually become a common pain point for young people. A total of 64.75% of college students actually sleep less than 8 hours a day, the proportion of sleep duration of more than 8 hours is only 7.97%, and the average sleep duration per day is 7.06 hours [5]. The sleep quality of Chinese college students is worrying, and it is urgent to improve the sleep quality of college students through intervention.

In future studies, researchers can carry out relevant scientific research work from behavioral intervention research (e.g. cognitive behavioral therapy [37]) or (e.g. sleep norms sleep hygiene development [38]). It is expected that more scholars can pay attention to the sleep health problems of college students.

### 4.2 College students’ physical exercise is mainly a small amount of exercise

The results of this study showed that the physical exercise of college students was mainly small (proportion: 76.118%), and the physical activity level of male students was better than that of female students. This finding may be attributed to the questionnaire collection period in this study, which was during the COVID-19 pandemic in China. At this time, the three surveyed schools adopted a “school closure” policy to restrict the spatial movement of students to cut off the transmission route of the virus [6], thereby minimizing the harm caused by COVID-19 to human health. According to previous studies, physical activity declined to varying degrees in different populations during the COVID-19 epidemic due to policy factors [39–42]. The overall decline in physical activity is also widespread in other Chinese universities [7, 43, 44]. The Chinese government’s COVID-19 prevention and control policy is “dynamic zero” [6] with the aim of ensuring people’s health, and policy factors may be an important reason for the decline in physical activity.

### 4.3 Excessive use of smartphones may be an important factor affecting the sleep quality of college students

The results of this study showed that smartphone use behavior was significantly negatively correlated with the sleep quality level of college students. In particular, the more active the mobile phone use behavior of college students, the worse their sleep quality. Previous studies have shown that excessive smartphone use can indeed lead to sleep disturbances and reduced sleep quality [45–48]. The “China Sleep Research Report (2022)” pointed out that the high use of smartphones by college students has become an important factor delaying the sleep time of college students and affecting their sleep quality [5]. The “China Sleep Research Report (2022)” pointed out that most universities survive the problem of “sleep delay,” with 27.52% of college students saying that they “always” sleep later than they expected, 26.80% of college students “sometimes” sleep later than they expected, and only 6.41% of college students almost never delay sleep. Many college students are dependent on mobile phones, resulting in sleeping extremely late.

From the analysis of the results of this study, the physical exercise level of college students may negatively affect their mobile phone addiction tendency. In particular, the higher the physical exercise level, the lower the college student’s mobile phone dependence addiction tendency. Studies have shown that people with more mobile phone users tend to reduce the chance of physical activity [49, 50], and sedentary behavior and calorie expenditure are also reduced [14, 51]. Therefore, college students’ “inactive” physical activity and higher risk of mobile phone addiction are likely to be causal, although more evidence is needed to prove this hypothesis. However, from the results of this study, the excessive use of smartphones may be an important factor affecting the sleep quality of college students.

### 4.4 Excessive use of smartphones can reduce the effect of physical exercise on sleep quality in college students

The results of this study show that smartphone use behavior plays a partial mediating role in the prediction of sleep quality by physical exercise in college students. As mentioned earlier, the excessive use of smartphones may be an important factor affecting the sleep quality of college students. Therefore, this study believes that excessive use of smartphones, combined with inactive physical activity behavior, is likely to increase students’ sleep quality problems. Possible reasons are as follows: First, excessive smartphone use has a significant negative effect on physical activity in college students, a result consistent with some previous views [24, 49]. Smartphones are the terminal medium for online social networking, app shopping, games, etc., and the abovementioned behaviors are recognized as static behaviors in front of the screen or sedentary behaviors [52]. As a result, excessive use of mobile phones reduces daily physical activity levels due to low levels of energy expenditure [53, 54]. Subsequently, physical activity levels can significantly predict sleep quality in college students, and low physical activity levels are an important cause of sleep quality in college students [55]. This scenario creates a “vicious circle” [56], in which the lower the level of physical activity of college students is, the more active their mobile phone use, which ultimately leads to poorer sleep quality.

However, there are also studies that have shown problematic social media use [PSMU] and problematic gaming [PG], unexpectedly demonstrated correlations with higher physical activity level [57]. This is quite different from the results of this study, and it is speculated that the possible reason is that college students have different purposes for using mobile phones. Some college students use smartphones to help practice sports-related sports, which is also a means to improve physical activity, especially based on current AI technology developments. The nature of these relationships warrants additional investigation into the underlying mechanisms in order to promote healthy lifestyles among university students.

### 4.5 Limitations

First, the measures of physical activity, sleep quality, and smartphone use behavior in this study used self-rated data, so recall bias may occur. Objective measurements can be developed with the help of wearable devices in subsequent studies to increase the objectivity of the measurements. Second, this study adopts a cross-sectional study design without longitudinal investigation across time periods and lacks strong historical evidence. Therefore, in future studies, the study design can be conducted from a longitudinal perspective to prove the causal relationship between variables more accurately and strongly. Finally, the PARS-3 and PSQI normal values used in this study are old, and MPATS has not yet established a data evaluation model for college students. The analysis of the data is mixed with continuous and categorical variables, which may also cause some errors in the interpretation of the results.

## 5 Conclusion

Chinese college students have poor sleep quality. Physical exercise and smartphone use behavior are important factors affecting the sleep quality of college students. Physical exercise can directly predict the sleep quality of college students and can predict the sleep quality of college students through the mediating effect of smartphone use behavior.
